# Delayed Recurrent Erythema Nodosum Following COVID-19 Vaccine: A Case Report

**DOI:** 10.7759/cureus.42776

**Published:** 2023-07-31

**Authors:** Nada Alghamdi, Rahmah M Alamrie, Anmar Y Alshafie, Serene R Almuhaidib

**Affiliations:** 1 Department of Dermatology, King Fahad University Hospital, Khobar, SAU; 2 Department of Dermatology, Dammam Medical Complex, Dammam, SAU; 3 Department of Medicine and Surgery, Imam Abdulrahman Bin Faisal University, Dammam, SAU

**Keywords:** dermatology, case report, pfizer-biontech vaccination, covid-19 vaccine, erythema nodosum

## Abstract

Erythema nodosum (EN) is a skin lesion that presents due to an inflammation of the subcutaneous fat, which is manifested clinically as a sudden onset of tender erythematous lesions. These lesions are typically localized to the pretibial surface. The cause of EN is mainly idiopathic, other causes are drugs, infections, autoimmune diseases, and inflammatory bowel disease. As vaccines are rarely known to cause EN, we are reporting a case of a 19-year-old female with a delayed recurrent reaction following the second dose of the COVID-19 vaccine.

## Introduction

Erythema nodosum (EN) is an inflammatory lesion of the skin, a form of panniculitis [1]. EN clinically manifested as a sudden onset of symmetrical tender erythematous nodules and plaques that are typically distributed bilaterally and involve the lower limbs mainly on the extensor surface but can also involve other areas such as the forearm, thighs, and ankles [2]. The etiology is idiopathic; however, it is found to be triggered by different possible factors that include infection, malignancy, inflammatory diseases, autoimmune diseases, pregnancy, and medications [3]. As vaccine-related EN is uncommonly seen [4], we report a case with delayed recurrent reaction following the second injection of the COVID-19 vaccine.

## Case presentation

This is a 19-year-old medically free single female who presented to the dermatology clinic through the emergency department with eye redness, joint pain, and tender erythematous lesions over the lower limbs that started one month prior to the presentation. The lesions began one month post her second dose of the Pfizer-BioNTech vaccine. The patient had a reaction that appeared 15 days after the first dose of the same vaccine. The reaction in both episodes was similar except that it was less severe in the second attack. She denied any recent or past history of infection.

**Figure 1:**
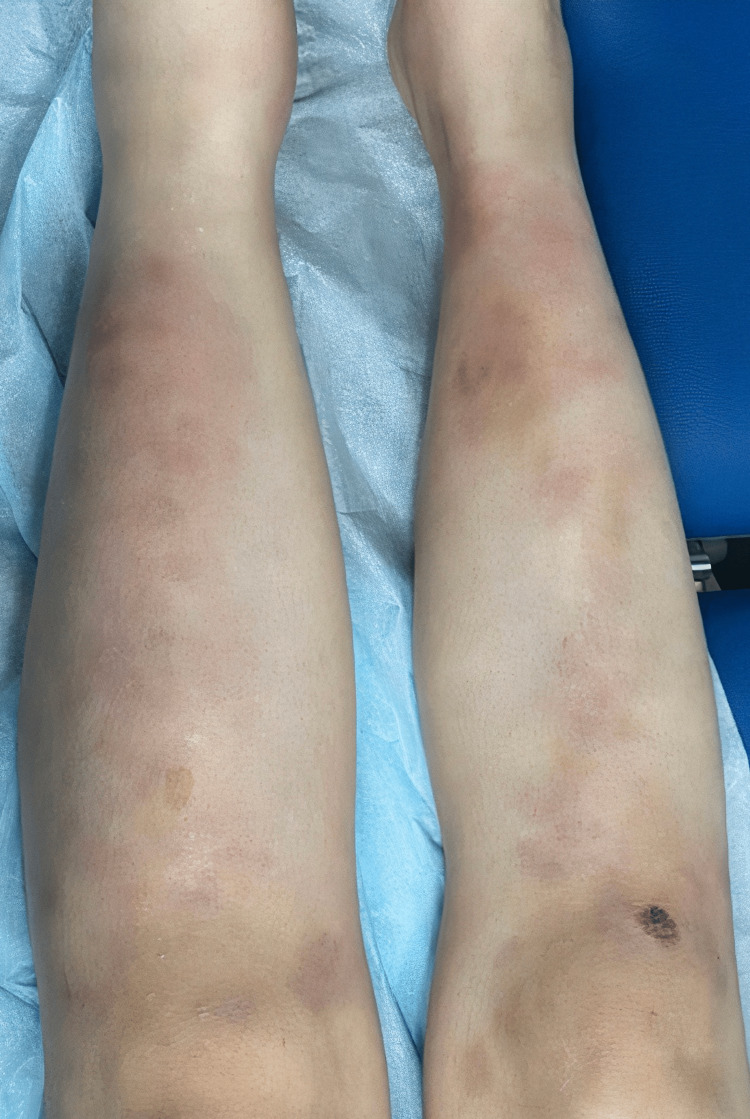
Multiple, tender, erythematous nodules ranging from 3 to 4 cm.

**Figure 2:**
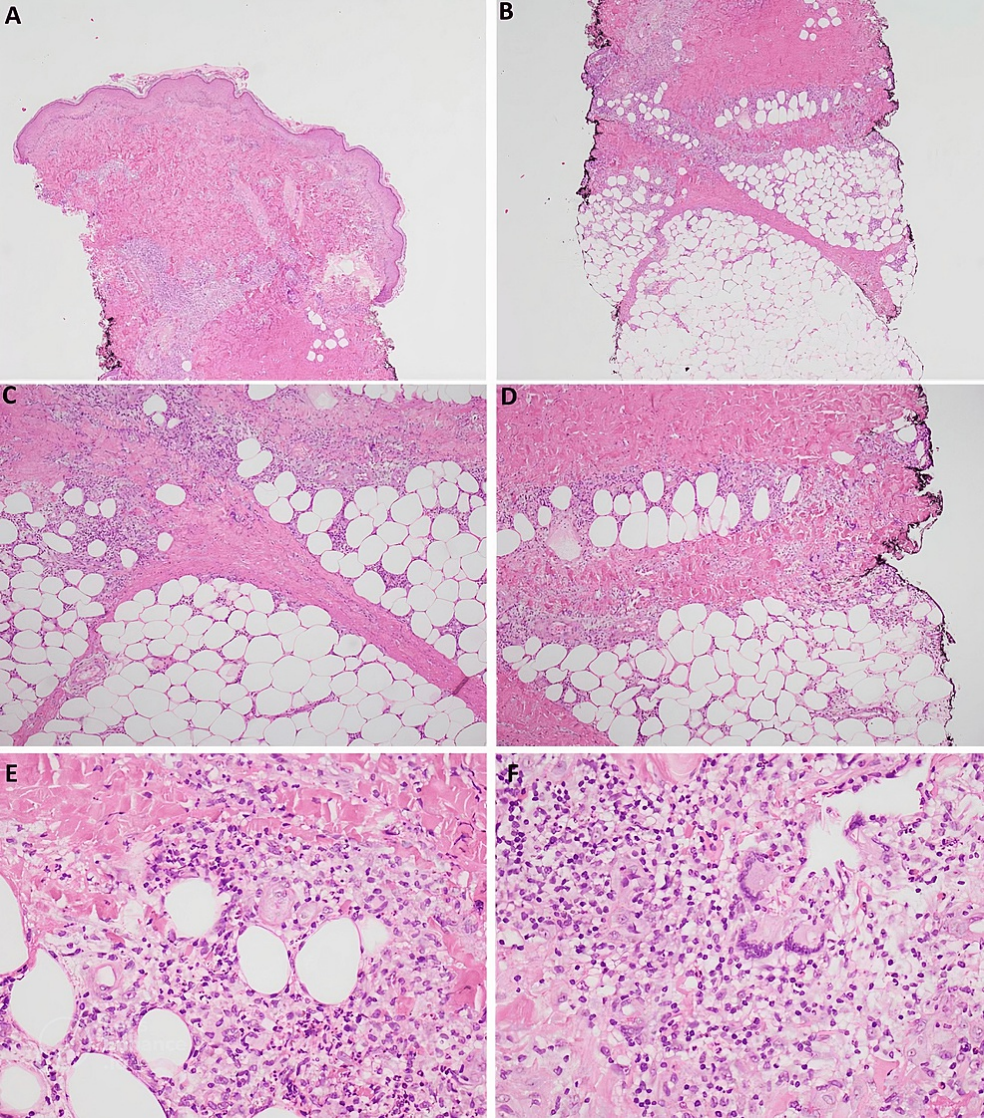
Skin biopsy shows epidermis which appears as basket wave stratum corneum, mild spongiosis, and intraepithelial lymphocytosis. The superficial dermis shows superficial and deep moderate perivascular mixed inflammatory cells infiltrate. The subcutaneous tissue shows predominantly septal mixed inflammatory cell infiltrate (lymphocytes, neutrophils, and eosinophils) along with giant cells suggesting an EN.

The examination was notable for bilateral conjunctivitis with superficial dilated vessels and no discharge. Additionally, she was found to have a lumbar spine, bilateral elbows, and knee tenderness. Lower limbs showed multiple, tender, erythematous nodules, with variable diameters ranging from 3 to 4 cm (Figure 1). There was no enlargement of the lymph nodes. Laboratory (Table 1) and radiology investigations were done that include complete blood count (CBC), transaminases, antistreptolysin O (ASO) titers, fecal culture, pregnancy test, hepatitis profile (types B and C), and chest X-ray. There were no abnormal findings, with an exception of an elevated erythrocyte sedimentation rate (ESR) and C-reactive protein (CRP), in addition to a positive test of Mycoplasma. A skin biopsy was done and was consistent with EN (Figure 2).

**Table 1:** Laboratory results.

Test	Result	Reference range
ESR	72 mm/hr	0 – 20 mm/hr
R-CRP	4.57 mg/dL	0.1 – 0.5 mg/dL
ASO titer	165 U/mL	< 200 U/mL
Fibrinogen	591 mg/dL	200 – 400 mg/dL
Mycoplasma IgM	13 U/L	≤ 0.76 U/L
Mycoplasma IgG	27 U/L	≤ 0.09 U/L
Urinalysis	++ RBCs	None
	++ Leukocytes	None

**Figure 3:**
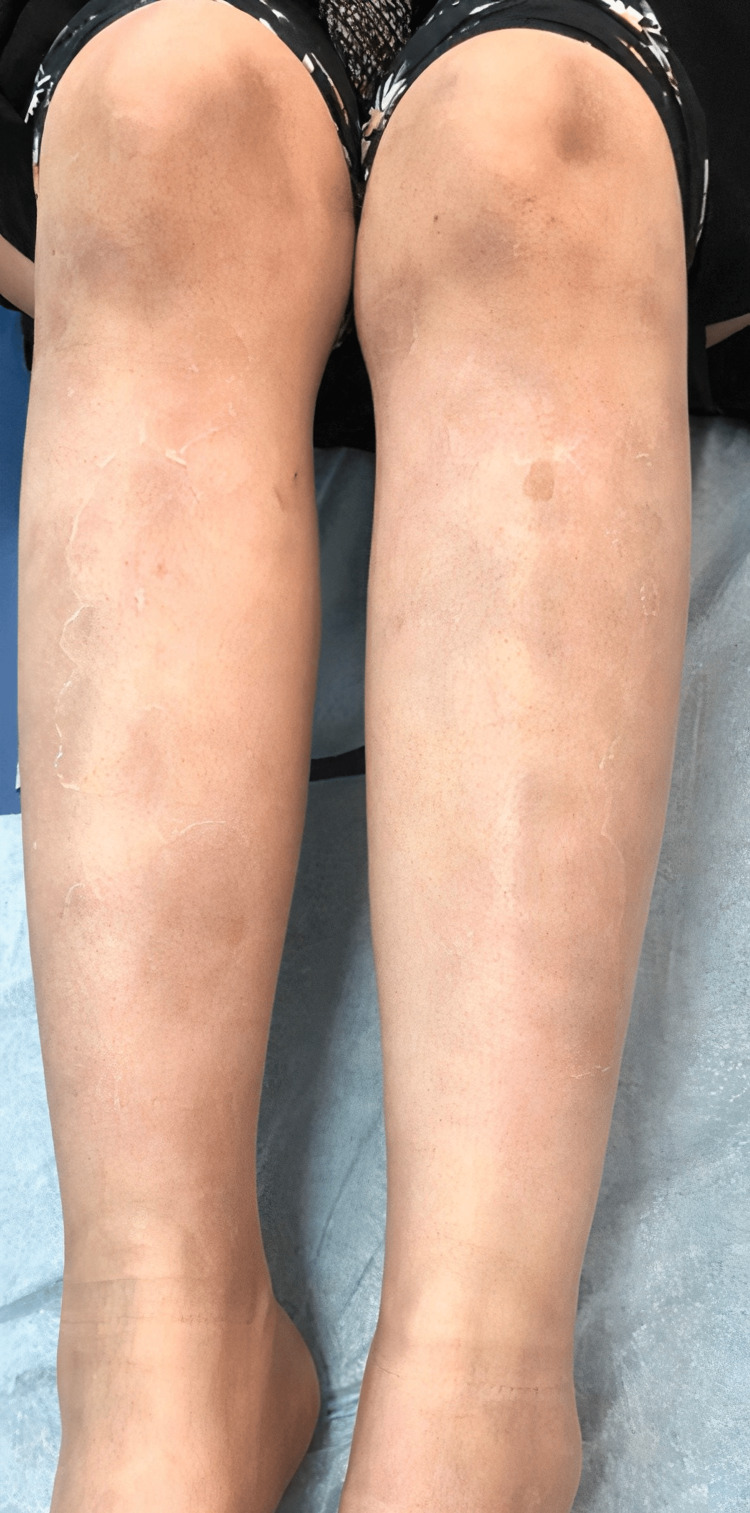
Post-inflammatory hyperpigmentation of the lower limbs.

The patient was started on symptomatic treatment that include oral corticosteroids, antihistamine, colchicine, and topical emollient. Two weeks post-presentation and initiation of treatment, the patient had a noticeable improvement in the cutaneous manifestations with post-inflammatory hyperpigmentation left behind (Figure 3).

## Discussion

Vaccines are biological preparation meant to enhance the immune system response to prevent infectious diseases; however, they can cause some side effects [5]. During the pandemic of COVID-19 infection, many types of COVID-19 vaccines were discovered, such as the Pfizer vaccine, AstraZeneca/Oxford vaccine, Moderna, and Johnson & Johnson vaccine [6]. The efficacy of the Pfizer vaccine/BNT162b2 against COVID-19 infection is 95% and was found to show some common local reactions such as redness, pain, and swelling [7]. In addition, some systemic reactions such as fever, headache, and fatigue range in severity from mild to moderate [7]. Many cutaneous reactions following COVID-19 vaccination were described in the literature, with delayed local reactions being the most common [8]. Stevens‐Johnson syndrome, toxic necrolysis syndrome, and anaphylaxis are the most severe [8]. Around two-thirds of these reactions were observed following the Moderna vaccine, and one-third following the Pfizer vaccine [8]. EN is a cutaneous inflammatory lesion that manifests clinically as a sudden onset of tender erythematous nodules and plaques, which are typically distributed bilaterally and involve the extensor surface of the lower limbs but can also involve other areas such as the forearm, thighs, and ankles [2].

The exact etiology is mainly idiopathic but has been reported to be triggered by different possible factors that include infections, malignancies, inflammatory diseases, autoimmune diseases, pregnancy, and medications [3]. In addition, vaccines are considered a rare cause of EN [4]. Eight vaccines were reported to be a possible cause of EN, and these are the hepatitis B vaccine, the Bacille-Calmette-Guerin vaccine, the malaria vaccine, the human papillomavirus vaccine, the smallpox vaccine, the rabies vaccine, the diphtheria, tetanus, and pertussis vaccine, and the cholera and typhoid vaccine [9-15]. EN can be diagnosed clinically, and a biopsy may also be done to confirm the diagnosis. Other underlying causes of EN need to be evaluated through a complete laboratory investigation that includes a CBC with ESR and CRP, ASO titers, a swab culture of the throat, and a chest X-ray [4].

## Conclusions

We report a case of EN that appeared after receiving the second dose of the Pfizer vaccine. More individuals are bound to receive COVID-19 and other types of vaccines worldwide. Therefore, physicians should be more aware of their possible cutaneous side effects to help achieve a faster diagnosis and prompt management.
